# Long-Term Clinical Experience With Metreleptin in a Brazilian Patient With Congenital Generalized Lipodystrophy Type 2

**DOI:** 10.1210/jcemcr/luaf185

**Published:** 2025-08-22

**Authors:** Isabella Sued Leão, Joana Rodrigues Dantas, Sarah Galvão, Melanie Rodacki, Lenita Zajdenverg

**Affiliations:** Medical Clinic Department, Nutrology and Diabetes Unit, Universidade Federal do Rio de Janeiro, Rio de Janeiro, 21941-913, Brazil; Medical Clinic Department, Nutrology and Diabetes Unit, Universidade Federal do Rio de Janeiro, Rio de Janeiro, 21941-913, Brazil; Medical Clinic Department, Endocrinology Unit, Universidade Federal do Rio de Janeiro, Rio de Janeiro, 21941-913, Brazil; Medical Clinic Department, Nutrology and Diabetes Unit, Universidade Federal do Rio de Janeiro, Rio de Janeiro, 21941-913, Brazil; Medical Clinic Department, Nutrology and Diabetes Unit, Universidade Federal do Rio de Janeiro, Rio de Janeiro, 21941-913, Brazil

**Keywords:** congenital generalized lipodystrophy, glycemic control, leptin replacement therapy, metabolic complications, metreleptin

## Abstract

We describe our 8-year clinical experience with metreleptin in a Brazilian adult female patient with congenital generalized lipodystrophy type 2 (due to a mutation in the *BSCL2* gene) and severe insulin resistance. The patient was initially treated with antidiabetic medications due to the unavailability of metreleptin. Metreleptin was initiated at age 20 years. Reductions from baseline for glycated hemoglobin (HbA1c) and triglycerides on metreleptin were sustained over a 5-year treatment period. The greatest reductions in HbA1c (from 10.8% [95 mmol/mol] to 6.0% [42 mmol/mol], −4.8%) and triglycerides (from 398 mg/dL [4.5 mmol/mL] to 104 mg/dL [1.2 mmol/L], −74%) occurred after 39 months, accompanied by a −95% decrease in total daily insulin usage (from 1600 to 88 IU/day). No significant adverse events occurred throughout metreleptin therapy. Metreleptin therapy was interrupted for 36 months due to limited access to the medication, during which time metabolic parameters deteriorated, returning to near-baseline levels. Thereafter, metreleptin was restarted. At the most recent clinic evaluation (3 months after resuming metreleptin), HbA1c, triglycerides, and liver enzyme levels reduced relative to the last measurements taken during treatment interruption. These findings provide support for the long-term and continuous use of metreleptin in patients with generalized lipodystrophy.

## Introduction

Congenital generalized lipodystrophy (CGL) is a rare, autosomal recessive disorder characterized by the near-complete loss of adipose tissue from birth or soon thereafter [1-3]. CGL is associated with severe insulin resistance, diabetes mellitus, hypertriglyceridemia, and leptin deficiency [2, 4]. Patients are at risk of developing cardiovascular, hepatic, and renal disease [2, 4]. Metreleptin, a recombinant analog of human leptin, was approved in Brazil in 2023 as an adjunct to diet as replacement therapy to treat the metabolic complications of leptin deficiency in patients aged ≥2 years with generalized lipodystrophy (GL), and in patients with partial lipodystrophy ≥12 years of age for whom standard treatments have not achieved adequate metabolic control [5]. The current multi-society guidelines for lipodystrophy recommend metreleptin as a first-line therapy (with diet) for the treatment of the metabolic and endocrine abnormalities of GL [4].

Here, we describe the long-term clinical outcomes (treatment duration, 8 years) from the first patient with CGL in our unit to receive metreleptin. When the patient started therapy, metreleptin was not registered in Brazil by the local health agency (Agência Nacional de Vigilância Sanitária) and was provided on a named-patient basis through local procedures. We also document the effects of metreleptin interruption (due to limited access) and resumption in this patient.

## Case Presentation

Our patient, a now 28-year-old woman with unrelated parents, was clinically diagnosed with CGL at age 18 months. Diagnosis was supported by a generalized loss of adipose tissue shortly after birth and the detection of hyperphagia during childhood. Complications associated with insulin resistance developed during late childhood, including diabetes mellitus (diagnosed at age 12 years). Metformin (along with an individualized meal plan) was initiated but was replaced 3 months later with insulin detemir plus aspart due to elevated liver enzymes. The development of severe insulin resistance led to progressive increases in antidiabetic medication doses. At age 15 years, an insulin pump was installed; the daily insulin dose was 9.6 IU/kg/day (basal-plus-bolus). Later, metformin (2 g/day) was restarted and was well-tolerated in combination with the insulin pump. Liver elastography (conducted at age 18 years) showed steatosis (assess using vibration controlled transient elastography) with evolution to severe fibrosis (elastography 14.3 kPa, interquartile range [IQR], 0.4 [3%]; controlled attenuation parameter [CAP] 341 dB/min, IQR 28; >66% hepatic steatosis; Meta-analysis of Histological Data in Viral Hepatitis [METAVIR] score, F4). Reference range values include normal liver stiffness below 5.5 kPa; values ≥7.1 kPa indicate significant fibrosis (corresponding to METAVIR stage F2 or higher); values ≥9.5 kPa suggest advanced fibrosis (≥ F3); and values ≥12.5 kPa are consistent with cirrhosis (METAVIR stage F4). These thresholds may vary slightly depending on the device and methodology used, but they are widely accepted in clinical practice in Brazil. For hepatic steatosis assessment using the CAP score, values between 260 to 263 dB/min are indicative of mild steatosis (≥ 5% hepatocyte involvement), 281 to 285 dB/min correspond to moderate steatosis (> 33%-66%), and 283 to 294 dB/min indicate severe steatosis (>66%). Dapagliflozin (10 mg/day) and pioglitazone (15 mg/day) were subsequently introduced in our patient. At age 19 years, the patient's pioglitazone dose was increased to 45 mg/day; however, no further improvement in glycemic control was observed.

**Figure 1.**
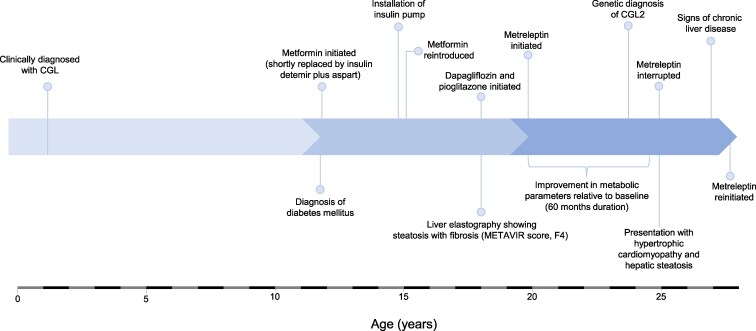
Chronological timeline of the medical evaluations and treatments.

Abbreviations: CGL2, congenital generalized lipodystrophy type 2; GL, generalized lipodystrophy; METAVIR, Meta-analysis of Histological Data in Viral Hepatitis.

Table 1 provides a history of the patient's glycemic profile and treatment prior to metreleptin. A timeline of the patient's medical evaluations and treatments is shown in Fig. 1.

**Table 1.** Glycemic parameters and treatment regimen prior to introduction of metreleptin

Visit number	Age of patient, years	HbA1c	Insulin, IU/day	Metformin	Dapagliflozin	Pioglitazone
1	18 years	12.6% (114 mmol/mol)	IP 700 IU	2 g/day	10 mg/day	—
2	18 years	10.5% (91 mmol/mol)	IP 1200 IU	2 g/day	10 mg/day	15 mg/day
3	19 years	9.5% (80 mmol/mol)	IP 1200 IU	2 g/day	10 mg/day	30 mg/day
4	19 years	10.6% (92 mmol/mol)	IP 1200 IU + 100 IU NPH (=1300 IU)	2 g/day	10 mg/day	45 mg/day
5	20 years	10.8% (95 mmol/mol)	IP 1200 IU + 200 IU NPH (twice/day) (= 1600 IU)	2 g/day	10 mg/day	45 mg/day

Abbreviations: HbA1c, glycated hemoglobin; IU, international units; NPH, neutral protamine Hagedorn.

## Diagnostic Assessment

Genetic testing for lipodystrophy using next-generation sequencing (Illumina® technologies) was conducted when the patient was approximately 24 years of age. This identified an autosomal homozygous recessive Chr11:62.694.680 G>T pathogenic variant of the *BSCL2* gene, confirming a diagnosis of CGL type 2 (CGL2).

## Treatment

Metreleptin was initiated as an adjunct to diet at age 20 years (5 mg/day), and the patient's antidiabetic regimen was maintained. Biochemical assessments conducted prior to metreleptin initiation showed elevated glycated hemoglobin (HbA1c) (10.8% or 95 mmol/mol) and triglyceride levels (398 mg/dL or 4.5 mmol/L), and moderately increased albuminuria (136.7 mg/dL) [Table 2, baseline visit].

**Table 2.** Clinical and laboratorial evolution during metreleptin treatment*^a^*

Visit number	Age of patient, years	Metreleptin dose	Duration of metreleptin treatment, months	HbA1c	TG	Insulin, IU/day	UAE	Weight
Baseline visit	20 years	—	0	10.8% (95 mmol/mol)	398 mg/dL	IP 1200UI + 200 IU NPH twice/day	136.7 mg/dL	62.5 kg
1	20 years	5.0 mg/day	2	8.2% (66 mmol/mol)	70 mg/dL	NPH 16 IU + regular 16 IU	—	—
2	20 years	7.5 mg/day	6	7.3% (56 mmol/mol)	186 mg/dL	NPH 48 IU + regular 26 IU	—	62.5 kg
3	20 years	7.5 mg/day	10	9.7% (83 mmol/mol)	199 mg/dL	IP 80 IU	—	63.0 kg
4	21 years	8.75 mg/day	16	9.0% (75 mmol/mol)	—	IP 100 IU	—	60.5 kg
5	22 years	10 mg/day	30	8.3% (67 mmol/mol)	116 mg/dL	NPH 8 IU + regular 72 IU	—	—
6	23 years	8.75 mg/day	39	6.0% (42 mmol/mol)	104 mg/dL	NPH 20 IU + regular 68 IU	—	58.5 kg
7	24 years	5.0 mg/day	57	9.0% (75 mmol/mol)	144 mg/dL	NPH 22 IU + regular 42 IU	—	61.0 kg
8	25	Interrupted	—	7.0% (53 mmol/mol)	145 mg/dL	NPH 24 IU + regular 46 IU	—	62.2 kg
9	25	Interrupted for 10 months	—	10.6% (92 mmol/mol)	—	NPH 54 IU + regular 52 IU	—	63.0 kg
10	27	Interrupted for 24 months	—	11% (97 mmol/mol)	230 mg/dL	NPH 450 IU + regular 300 IU	49.3 mg/dL	64.0 kg
11	28	10 mg/day	3 months after restarting	8.1% (65 mmol/mol)	190 mg/dL	NPH 120 IU + aspart 84 IU	—	63.0 kg

Abbreviations: HbA1c, glycated hemoglobin; IP, insulin pump; IU, international units; NPH, neutral protamine Hagedorn; TG, triglycerides, UAE, urinary albumin excretion; –, not measured.

^*a*^Oral antidiabetic medication was continued during treatment with metreleptin: pioglitazone, dapagliflozin, and metformin.

Two months after starting metreleptin, there was an improvement in glycemic control, leading to a reduction in her total daily insulin dose. Her insulin pump was removed, and an antidiabetic regimen of 16 IU/day neutral protamine Hagedorn (NPH) insulin (0.25 IU/kg) and oral dapagliflozin and pioglitazone was retained (Table 2, visit 1). After 6 months of metreleptin (Table 2, visit 2), HbA1c and triglyceride levels had reduced to 7.3% (56 mmol/mol) and 186 mg/dL (2.1 mmol/L), respectively.

Improvements in hyperphagia, liver pathology, and hepatic steatosis were observed after 6 months of metreleptin (elastography 15.5 kPa, IQR 3.1 [20%]; CAP, 285 dB/min, IQR 40; 11%-33% steatosis; METAVIR score, F4) together with normalization of transaminases, canalicular enzymes, and albuminuria. Amelioration of hypertrophic cardiomyopathy was recorded, with ventral septum thickness regressing to 12 mm (on echocardiogram; the reference value for women in Brazil is 9.3 mm).

An increase to the maximum metreleptin dose (10.0 mg/day) was required after 16 months (ie, after visit 4, Table 2), due to limited improvements in glycemic control. At this time, the patient reported poor adherence to insulin treatment. The development of anti-metreleptin antibodies was considered to contribute to this reduced glycemic control; however, specific tests were not conducted, and results remain inconclusive. Her metreleptin dose was later readjusted to 8.75 mg/day (Table 2, visit 6).

Overall, reductions in HbA1c and triglycerides, relative to baseline, were observed at all follow-up visits over a 60-month treatment period. The greatest reduction in HbA1c from baseline (from 10.8% [95 mmol/mol] to 6.0% [42 mmol/mol]; −4.8% [corresponding to a change of −29 mmol/mol]) was observed after 39 months of therapy and was accompanied by a decrease of 95% in total daily insulin (from 1600 to 88 IU/day; Table 2, visit 6). A −74% decrease in triglycerides from baseline (based on mg/dL values) also occurred at this time point (from 398 mg/dL [4.5 mmol/L] to 104 mg/dL [1.2 mmol/L]).

During the coronavirus disease 2019 (COVID-19) pandemic, virtual consultations were conducted without laboratory tests, and the patient reported satisfactory glycemic control based on self-monitoring of blood glucose (SMBG).

## Outcome and Follow-Up

Limited access to metreleptin necessitated a dose reduction from 8.75 mg/day to 5 mg/day after 57 months (Table 2, visit 7). Three months later, metreleptin was interrupted (Table 2, visit 8). The patient did not experience any serious adverse events related to metreleptin during the 60-month treatment period. The greatest reduction in weight loss relative to baseline was recorded 39 months after initiation of metreleptin (−4.0 kg) [Table 2, visit 6].

Approximately 10 months after interruption of metreleptin, the patient returned to the clinic and presented with elevated HbA1c (10.6%; 92 mmol/mol; Table 2, visit 9). Insulin doses were increased, the basal-plus-bolus regimen was reintroduced, and an insulin pump was reinstalled. Subsequently, the patient presented with hypertrophic cardiomyopathy (ventricular septum thickness of 19 mm on echocardiogram) without symptoms, hepatic steatosis with evolution to severe cirrhosis, and primary amenorrhea without the other phenotypic features of Turner syndrome. Previously, at the age of 18, transdermal estradiol was recommended, but the patient declined treatment.

A dual-energy x-ray absorptiometry scan conducted ∼24 months following the interruption of metreleptin measured total body fat percentage at 8.2% with complete absence of visceral fat. Recurrence of albuminuria (49.3 mg/dL), with further increases in HbA1c (11.0% or 97 mmol/mol) and triglycerides (230 mg/dL or 2.6 mmol/L) observed (Table 2, visit 10). Liver enzymes were also elevated at this time: alanine aminotransferase (ALT), 100 U/L; aspartate aminotransferase (AST), 106 U/L; gamma-glutamyl transferase (GGT), 228 U/L; alkaline phosphatase (ALP), 170 U/L. Ultrasonography of the upper abdomen showed signs of chronic liver disease, marked steatosis, splenomegaly, and increased portal vein caliber with hepatic arterialization. Liver elastography corroborated these findings (elastography 24.3 kPa, IQR 2.6 [11%]; CAP 307 dB/min, IQR 12; >66% steatosis; METAVIR score, F4). Upper gastrointestinal endoscopy revealed portal hypertensive gastropathy without esophageal varices.

Following a 36-month period of interruption, metreleptin was resumed at age 28 years. At the most recent follow-up clinic visit (ie, 3 months after restarting metreleptin), the patient was receiving 10 mg of metreleptin per day and had decreases in HbA1c (8.1% or 65 mmol/mol, leading to a reduction in insulin dose) and triglycerides (190 mg/dL or 2.2 mmol/L) relative to the last measurements taken during the period of metreleptin interruption (Table 2, visit 11). Ultrasound of the upper abdomen showed signs of chronic liver disease and portal hypertension, but with reduction of steatosis to mild (previously marked). Improvements in liver enzyme levels were also recorded (ALT, 43 U/L; AST, 45 U/L; GGT, 62 U/L; ALP, 190 U/L).

## Discussion

We present our long-term clinical experience of metreleptin in a patient with CGL2. Our patient showed an early response to metreleptin (after 6 months) that was sustained over a 5-year treatment period.

The clinical program for metreleptin demonstrated the efficacy of metreleptin in patients with acquired and genetic forms of GL, in which significant mean reductions from baseline for HbA1c, fasting plasma glucose, and triglycerides were sustained over 36 months [6]. Furthermore, the effect of metreleptin on hepatic steatosis has been reported following evaluation of liver biopsies from 27 individuals with lipodystrophy [7]. This analysis showed that 33% of patients had borderline or definite nonalcoholic steatohepatitis (NASH) after 26 months of metreleptin compared with 86% of patients prior to metreleptin [7]. These improvements were accompanied by mean reductions in ballooning injury and nonalcoholic fatty liver disease activity score [7].

Real-world evidence also supports the long-term effectiveness of metreleptin in GL. For example, a case series analysis involving 5 CGL2 cases from Spain who received metreleptin (over a treatment period ranging from 21 to 63 months) reported reductions from baseline for HbA1c (of up to −7.5% or −58 mmol/mol) and triglycerides (of up to −90%) [8]. Amelioration of high-density lipoprotein-cholesterol (HDL-c), insulin sensitivity, and hepatic transaminases were also documented in most of these patients [8]. Consistent with these findings, our patient showed a reduction in HbA1c (−4.8%) and triglycerides (−74%) and a −95% decrease in daily insulin usage relative to baseline after 39 months of metreleptin.

Notably, our patient experienced a worsening of their metabolic disease 10 months after metreleptin therapy was interrupted. Following 24 months of metreleptin interruption, HbA1c and triglyceride levels had returned to near-baseline values. Worsening liver complications (ie, marked steatosis and elevated liver enzymes) were also recorded during this period. The first clinical study of metreleptin in lipodystrophy showed that withdrawal of metreleptin resulted in an increase in fasting triglycerides and glycemic parameters within 48 hours; reinitiation of metreleptin normalized these parameters [9]. Restarting metreleptin also improved the metabolic complications that had deteriorated over a 9-month treatment discontinuation period in a patient with acquired generalized lipodystrophy [10]. Similarly, the data from our patient illustrate the metabolic effects of metreleptin interruption and how HbA1c, triglyceride levels, hepatic steatosis, and liver enzymes may improve following metreleptin resumption.

Although not assessed by us, it is plausible that impaired beta-cell function may have developed in our patient prior to metreleptin initiation and contributed to the metabolic deterioration that followed metreleptin interruption. In support of this, a recent retrospective study showed that in patients with GL who initiated metreleptin before the onset of severe metabolic complications had better long-term control of diabetes, proteinuria, hypertriglyceridemia, and liver enzyme levels vs those who started therapy after severe metabolic complications had developed [11].

In conclusion, we describe sustained metabolic response to metreleptin over a 5-year period in a patient with CGL2. Interruption of metreleptin resulted in a return of metabolic parameters to near-baseline values within a period of 24 months. Clinical improvements observed 3 months following metreleptin resumption provide support for the long-term and continuous use of metreleptin in GL.

## Learning Points

- In the absence of a treatment that directly targets leptin deficiency, metabolic disease in CGL2 progresses, leading to severe, multisystem complications.
- Our CGL2 case showed a sustained response to metreleptin therapy over a 5-year treatment period with no new safety signals. During this time, her daily insulin dose and HbA1c reduced by a maximum of −95% and −4.8% relative to baseline (at month 39), respectively.
- Our CGL2 case highlights how interruption of metreleptin can lead to significant deterioration in metabolic outcomes. Reinitiation of metreleptin led to improvements in glycemic and lipid parameters, reduction in liver steatosis, and normalization of liver enzymes. These results support the long-term and continuous use of metreleptin in patients with generalized lipodystrophy.

## Data Availability

Original data generated and analyzed for this case report are included in this published article.

## Abbreviations

ALT: alanine aminotransferase
ALP: alkaline phosphatase
AST: aspartate aminotransferase
CAP: controlled attenuation parameter
CGL: congenital generalized lipodystrophy
GGT: gamma-glutamyl transferase
GL: generalized lipodystrophy
HbA1c: glycated hemoglobin
IQR: interquartile range
METAVIR: Meta-analysis of Histological Data in Viral Hepatitis
